# Each the Best

**Published:** 1868-08

**Authors:** 


### EACH THE BEST.

A GENTLEMAN called for medical advice the other day, whose reputation as the manufacturer of a certain article of hardware would sell any thing with his name upon it. On being asked if the quality of the article depended on any secret mode of preparation, or any peculiarity of handwork, or any patent protection, he replied: "Nothing of the sort; the article is not patented, nor is there any secret in my establishment. On the contrary, while my process is peculiar, it has at the same time been made public, and I have debated the propriety of my course, and defended it against my friends and rivals in the same business. I took my stand at the age of twenty years, and have resolutely adhered to it for nearly a third of a century through every phase of the market; there is no year, of all that time, in which we failed to make money; although, at times, we may have been compelled to sell at actual cost. But now, for many years, I have had the preference in the markets of the world; seeing this, my name was pirated by thirty manufacturers in Manchester, at one and the same time, and in every single case, the English courts decided in my favor, and compelled the rectification of the wrong. My plan was simply this; I allowed my name to be stamped, only on the very best article my establishment could turn out. I had a finished expert in each particular branch and stage of the manufacture, to inspect the article; he had nothing else to do, and it was not 'passed,' until each one had declared that it was without a flaw. At first, multitudes were thrown aside, but by rigidly requiring each department to strive to make each successive article the best of any that preceded it, we now habitually make '*each the best*,' and this is the answer to your question." So he will live longer, who will strive to make each day the best, in industrious exercise and a comprehensive temperance; who, if he work too little to-day for a good night's rest, will strive to work more to-morrow; if he eats too much to-day for subsequent comfort, will retrench to-morrow, or so modify his eating in quantity or quality, that there will be no unpleasant reminder after any meal, that an excess has been committed; this apparently little thing has engaged the attention, and waked up the resolves, and baffled the efforts of the greatest minds in all ages; showing that a brute appetite wars daily with a nature divine, as, in handicraft, cupidity tempts integrity and generally overcomes it; hence so few men, in any branch of business, legitimately succeed in the long run. Let every nurse, then, endeavor to do better with each succeeding invalid; let every physician endeavor to give more thought and attention to each new patient, and let us all, in all things, strive patiently, resolutely, persistently, to make each day "the best" of any that preceded it, and thus do more than could possibly be done in any other way, to prevent our lives being a failure; a calamity than which no greater could befall any man. Reader! let you and I strive to live, so that it may be justly inscribed on the slab which covers our graves:

"HE DID NOT LIVE IN VAIN."

